# A Structural Equation Modeling of the Relationships between Depression, Drug Abuse and Social Support with Suicidal Ideation among Soldiers in Iran in 2015

**Published:** 2016-11-22

**Authors:** Mehdi Nosratabadi, Zohreh Halvaiepour

**Affiliations:** ^a^ Social Determinants of Health Research Center, Isfahan University of Medical Sciences, Isfahan, Iran; ^b^ Faculty of Education and Psychology, University of Isfahan, Isfahan, Iran

**Keywords:** Suicide, Drug Abuse, Depression, Social Support

## Abstract

**Background:** Military service is a crucial period in the lives of young people and during this period soldier facing with multiple psychosocial problems.

**Objectives:** The present study aimed to explore structural analysis of the relationships between depression, drug abuse, social support and the risk of suicidal ideation among Military Medical University soldiers in Iran.

**Methods:** In the present correlational research, a sample of 176 soldiers, from three units, was selected using randomly stratified sampling. Data were collected through the Social Support Questionnaire (SSQ), the Beck Depression Inventory-II (BDI-II), the Beck Scale for Suicide Ideation (BSS) and the Possibility of Drug Abuse Scale (LDAS). Structural equation modeling was used to test the fit of the model, identify direct and indirect effects of the psychosocial correlates. Data were analyzed using the SPSS and AMOS software (Verson22).

**Results:** out of the whole subjects, 28.4% had suicidal ideation and 65.3% had degrees of depression (mild to severe). A significant reverse relationship was observed between social support and suicidal ideation (p<0.05). The strongest relationship was detected between drug abuse and suicidal ideation. The final structural model indicated that 74% of the variance in suicidal ideation was explained by the three examined variables of depression, social support and drug abuse.

**Conclusions:** The overall results showed that the risk of suicidal ideation, depression and drug abuse are relatively significant in Military Medical University soldiers requiring taking serious actions by the authorities and other relevant organizations in order to improve the psychosocial health status of these soldiers.

## Introduction

Military service is a crucial period in the lives of young people. Facing with new roles, intensive trainings and many other issues can be considered as sources of stress in this period; thus, the possibility of the formation of psychosocial damages will be high if soldiers do not adapt themselves to the existing situations. Committing suicide is among the most important psychosocial damages in this period, which results from multiple family-based and social factors along with drug abuse and depression^1,2^.

Suicidal ideation is defined as self-reported thoughts about suicide beginning from a vague desire to die and ending in an attempt to commit suicide ^3^. According to the estimations provided by the WHO, the rate of suicide death has reached to one every 20 seconds and one person commits suicide each 1 or 2 seconds^4^. The frequency of successful suicide among military people is 12/100,000 people and among civilians is 9/100,000 ^5^.

According to the WHO, 3.3 million people die annually due to drug abuse and alcoholism, and in the age group 20 – 39 yr approximately 25% of the total deaths are alcohol-attributable ^6^. There are different reasons for addiction in adolescents among which detachment from family and economic status can be mentioned ^7^. Although drug abuse and alcoholism are illegal among military personnel, studies have shown high rates of drug abuse and alcoholism among the military forces of countries around the world ^8^. 20.8% of the Iranian youth who enter active duty military service are constant users of cigarettes and 72.4% have smoking experience ^9^. Studies on people who have committed suicide have shown that 19 to 63% of them are suffering from one of the drug abuse-related disorders ^10^. In Iran, drug abuse (with the ratio of 0.54) is the second most common disorder in people committed suicide or people with a history of suicide attempt ^11^.

In 90% of suicides, psychiatric disorders are involved and depression is among the highest risk factor that appears to be comorbid with suicidal ideation ^12^. Previous studies have concentrated on the role of depression, as an explanatory variable, in drug abuse and suicidal ideation^13,14^. Depression is an important factor in 50% of suicides among soldiers ^15^.

Social support is a multidimensional construct indicating individuals’ perceptions of being supported, loved and valued, belonging to communication networks and considering mutual obligations towards other people are among the features of social support ^16^. Social support is a protective factor against suicide in the general population and among soldiers ^17^.

Given the importance of soldiers’ psychosocial health status, the present study was conducted to explain the relationships between depression, drug abuse, social support and the risk of suicidal ideation among Military Medical University soldiers in Iran. The present study aimed to explore structural analysis of the relationships between depression, drug abuse, social support and the risk of suicidal ideation among Military Medical University soldiers in Iran

## Methods

This study was conducted based on the structural equation modeling. Hair et al., ^18^ recommend a sample size of at least 100 observations to achieve adequate power in structural equation modeling. Besides, most studies recommend using sample sizes of at least 200/ 5 or 10 cases per parameters ^19,20^, thus in the current study 200 soldiers were recruited. The final sample consisted of a 176 soldier students who agreed to participate and returned valid questionnaires (24 questionnaires were not valid). They were recruited using randomly stratified sampling from three units, including hospital, security unit and administrative affairs.

The study was approved by the Department Research Review Committee of Aja‏ University‏ of‏ Medical Sciences (number-993457).

Four questionnaires were employed to collect data as follows:

### The Beck Scale for Suicide Ideation (BSS)

The BSS is a 19-item self-report questionnaire scored on a three-point Likert scale ranging from 0 to 2; thus, total scores range from 0 to 38. In the BSS, scores in the range of 0-5 indicate absence of specific plans for suicide; scores in the range of 6-19 indicate readiness for suicide; and scores in the range of 20-38 indicate specific plans for suicide. Cronbach’s alpha (internal consistency) as well as concurrent reliability of this scale has been reported as 0.89-0.96 and 0.83 respectively. This scale is significantly correlated with the Beck’s scales for depression and hopelessness ^21^, validated on Iranian soldiers^22^. A Cronnach’s alpha of 0.95 and a concurrent validity of 0.76 with the depression subscale of General Health Questionnaire is reported.

### The Probability of Drug Abuse questionnaire

This 16-item questionnaire was first introduced by Pour Sharifi et al^23^ in 2005. Individuals indicate how much they engage in listed activities regarding drug abuse and smoking in month ago. This scale consists of 17 items and is rated on 4-point scale ranging from 1 (at all) to 4 (always). Scale has been developed by review of valid sources regarding people’s vulnerability to drug abuse. In this questionnaire, each item represents a risk factor and more risk factors indicate higher possibility of drug abuse. Regarding the highest component loading in factor analysis, one item is selected as a core item ratio of each question against it is computed. Finally, the total score is constructed by summing up the weighted average of each item. This scale has been reliable and valid in Iranian student samples ^24,25^.In our study, the Cronbach’s alpha of the scale was 0.81.

### The Beck Depression Inventory-II (BDI-II)

The BDI-II is the revised version of the Beck Depression Inventory (BDI) that measures the severity of depression in people. This scale is developed in line with the Diagnostic and Statistical Manual of Mental Disorders (Fourth Edition) (DSM-IV) criteria for depression. The BDI-II includes 21 groups of items and each group consists of four options. The total scores range from 0 to 63; accordingly, in this study, those scored below 10 were considered as healthy and those scored above 10 were considered as people with degrees of depression. The Cronbach’s alpha and test-retest reliability of this scale have been reported as 0.87 and 0.74 respectively^26,27^.

### The Social Support Questionnaire (SSQ)

The 4-item SSQ, used in this study, was extracted from the original SSQ provided earlier ^28^. To obtain the total score of this questionnaire, a factor analysis was conducted and all items formed one factor on this basis, 1 point was considered for each positive answer and no point for negative ones. Due to the proximity of the weight and importance of the items weighting were not considered and the sum of all points considered as the total score of the SSQ. Thus, the total score of the SSQ ranged from 0 to 4. The obtained Cronbach’s alpha of the SSQ was 0.81.

### Data analysis

We performed statistical analysis via SPSS version 20.0 (Chicago, IL, USA). We constructed and tested a structural equation model- path analysis with latent variables (SEM) using AMOS module 20.0 to interpret suicidal ideation among soldiers. SEM is advantageous in identifying both direct and indirect effects, as well as measuring the overall model fit.

Several indices to assess the fit of the model (the ability of the model to reproduce the data) were used as follows: The Root Mean Square Standard Error of Approximation (RMSEA), the Goodness-of-fit statistic (GFI), and the adjusted goodness-of-fit statistic (AGFI). For RMSEA, values of approximately 0.06 or less indicate adequate fit, For both the GFI and AGFI, a value more than 0.75 is consistent with a good model fit^29^. The threshold for statistical significance was set at P<.05. Finally, structural models were fitted to discover the direct and indirect effects.

## Results

Out of 176 soldiers, 157 (89.8%) were single; 151 people were under the age of 23 yr and 67 people (38.1%) had finished guidance school. In order to reporting drug abuse among soldiers, the median of drug abuse (number 83) along with one level below and one level above it were considered. Accordingly, soldiers in the above-the-median group were more likely drug abusers. Out of 92 soldiers in the above-the-median group, 81.1% were under the age of 23 yr and 18.5% were older. 65.3% of the soldiers were suffering from degrees of depression (mild to severe).

Exploratory Factor Analysis was applied in factor analysis to extract the valid items for BSS, Probability of Drug Abuse Scale and BDI-II. Based on the selected items, the Kaisere Meyere Olkin (KMO) measure of sampling value for BSS, Probability of Drug Abuse Scale and BDI-II were 0.77, 0.76 and 0.70 respectively. The reliability of all extracted items was confirmed through Cronbach's alpha coefficients that were higher than 0.70, as mentioned earlier.

In Table 1, the prevalence of suicidal ideation among soldiers is presented based on their age. Accordingly, out of the 50 soldiers (28.4%) with high risk of suicidal ideation, 31.1% were under 23 yr and out of the 126 soldiers with suicidal ideation, 68.9% were under 23 yr.

**Table1:** The prevalence of suicidal ideation among soldiers in terms of their age

**Age group (yr)**	**With suicidal ideation**		**Without suicidal ideation**		**Total**	
	**Number**	**Percent**	**Number**	**Percent**	**Number**	**Percent**
≤23	104	68.9	47	31.1	151	100
>23	22	88.0	3	12.0	25	100
Total	126	71.6	50	28.4	176	100

To investigate the relationships between suicidal ideation and depression, drug abuse and social support, correlation coefficients were used. There were positive significant relationships between suicidal ideation and the variables of drug abuse (r =0.63, *P*<0.05) and depression (r =0.65, *P*<0.05) indicating that higher levels of depression as well as drug abuse increased suicidal ideation in the examined soldiers. There was also a negative significant relationship between social support and suicidal ideation (r =-0.26, *P*<0.05) so that soldiers who were more socially supported showed lower levels of suicidal ideation.

Table 2 shows parameters for explaining suicidal ideation in soldiers based on the variables of depression, drug abuse and social support. Accordingly, squared multiple correlations for suicidal ideation was 0.74. In other words, 74% of the variance in suicidal ideation among soldiers is attributed to the three mentioned variables. Squared multiple correlations of drug abuse (as an intermediate variable) is 0.28, representing 28% of the variance in drug abuse was explained by the variables of depression and social support. Squared multiple correlations of depression is 0.22. In other words, 22% of the variance in depression was explained by the variable of social support. As indicated in Table 2, the effect of social support on drug abuse was not significant (*P*>0.05). In other words, social support had no direct effect on drug abuse but affected it indirectly (regression coefficient of -0.24). Similarly, social support had no direct effect on suicidal ideation but affected it indirectly (regression coefficient of -0.25). The indices of goodness of fit in this model were estimated as satisfying (RMSEA=0.06, AGFI=0.75, GFI=0.79).

**Table 2:** Standardized regression coefficients (β), Critical Ratio (CR), P-values, Standard error (S.E) direct and indirect effects of each factor in the model

**Variables**	**Coefficient (β)**	**Critical ratio (CR)**	**P value**	**SE**	**Direct effect**	**Indirect effect**
Drug abuse on suicidal ideation	0.67	2.5	0.010	0. 26	0.67	-0.26
Depression on suicidal ideation	0.64	4.4	0.000	0.14	0.28	0.36
Depression on drug abuse	0.55	2.4	0.010	0.22	0.53	-
Social support on drug abuse	-0.18	0.8	0.400	0.22	0.06	-0.24
Social support on depression	-0.46	-5.8	0.000	0.07	-0.46	-
Social support on suicidal ideation	-0.25	-3.6	0.010	0.06	-	-0.25

## Discussion

The present study was conducted to explain the relationships between depression, drug abuse and social support and the risk of suicidal ideation among Military Medical University soldiers. The final structural model indicated that 74% of the variance in suicidal ideation was explained by the three examined variables of depression, social support and drug abuse. According to this model, depression has a direct effect on suicidal ideation and affects suicidal ideation through the variable of drug abuse. In both cases, higher levels of depression lead to higher rates of suicidal ideation and drug abuse. This finding was in line with the results of another study ^13^ that focused on the role of depression, as an explanatory variable, in psychosocial disorders such as drug abuse and suicidal ideation.

In this model, 28% of the variance in drug abuse can be explained by the variables of social support and depression. Drug abuse can have a mediator role in the relationship between social support and suicidal ideation^30,31^. It seems that interaction with family members, friends and acquaintances can be effective in reducing the possibility of drug abuse and suicidal ideation in soldiers. To explain this, it can be stated that soldiers who have experienced less social support are suffering from multiple problems. In other words, these soldiers are less satisfied with their lives and have less social interactions, their self-confidence is lower and they may feel worthless. It is obvious that these issues make them vulnerable to drug abuse. Therefore, it seems that the most possible reaction of these soldiers to their multiple problems is drug abuse that provides an immediate response to their existing situation. In this study, social support was not directly related to suicidal ideation; however, it was associated with suicidal ideation through the variable of depression. This finding was consistent with the findings of other studies^32,33^ showing that greater social support can protect people from the development of depression and traumatic stress symptoms. Thus, social support can acts as a psychosocial buffering of suicidal ideation. Social support is associated with reduced depressive symptoms in soldiers by reducing psychological stresses and increasing social interactions^34^.

Drug abuse in interacting with psychological and social factors (e.g. depression and social support), can better explain suicidal ideation in soldiers. In an analysis of predisposing factors for suicide in soldiers, Nouri and colleagues showed the relationships between family support, mental disorders and addiction with suicide ideation^35^.

Our results showed that the risk of suicidal ideation is relatively significant in Military Medical University soldiers requiring serious actions taken by the authorities and other relevant organizations in order to improve the psychosocial health status of these soldiers. Preventive actions to improve soldiers’ conditions make the military service period a pleasant event in their lives. Since depression is considered a major risk factor for drug abuse and suicidal ideation, it is recommended to monitor soldiers’ psychological profiles periodically and provide adequate psychological care accordingly (especially for younger soldiers and those who live far from their families). In this study, the strongest association was revealed between drug abuse and suicidal ideation; thus, preventive measures to stop the distribution and use of tobacco (as a gateway to drug abuse) must be taken at army barracks. Moreover, providing training courses related to high-risk behaviors of drug abuse (specifically new substances) for soldiers is actually a need.

Among the limitations of the present study, findings based on self-report data and lack of soldiers’ full cooperation in the completion of the questionnaires can be mentioned. Therefore, it is recommended that the units’ commanders and soldiers cooperate more with the future researchers.

## Acknowledgments

We would like to appreciate all the participants and others who helped us in this research.

## Conflict of interest statement

The authors declare there are no competing interests‏.

## Highlights

- This study suggests positive significant relationships between suicidal ideation, drug abuse and depression in soldiers.
- In Iranian soldiers, drug abuse can act as a mediator in the relationship between social support and suicidal ideation.
- Risk of suicidal ideation is relatively significant in Military Medical University soldiers requiring serious actions taken by the authorities.
